# Influence of Denture Base Fabrication on *Candida albicans* Adhesion and Early Biofilm: An In Vitro Comparison of Five Techniques

**DOI:** 10.3390/dj14050262

**Published:** 2026-05-01

**Authors:** Victor Moreno-Prieto, Carlos Enrique Guillén-Galarza, Christian Esteban Gómez-Carrión, Ignacio Schwan-Silva

**Affiliations:** Facultad de Ciencias de la Salud, Escuela de Odontología, Universidad Norbert Wiener, Av. Arequipa 444, Lima 15046, Peru; victor.moreno@uwiener.edu.pe (V.M.-P.); carlos.guillen@uwiener.edu.pe (C.E.G.-G.); christian.gomez@uwiener.edu.pe (C.E.G.-C.)

**Keywords:** *candida albicans*, denture bases, biofilms, polymethyl methacrylate, good health and well-being

## Abstract

**Background/Objectives:** Denture stomatitis is closely associated with Candida albicans colonization of denture-base surfaces. This in vitro study compared early adhesion (1 h) and initial biofilm formation (24 h) of *C. albicans* across five denture-base-related material groups using adhered cell counts and adhered/inoculum proportions. **Methods:** A 5 × 2 factorial design (five material groups; 1 and 24 h) evaluated a comparator pattern resin, heat-polymerized acrylic resin, autopolymerizing acrylic resin, milled CAD/CAM PMMA, and microwave-polymerized acrylic resin. All specimens underwent standardized finishing and mechanical polishing before microbiological testing. Data were log10-transformed and analyzed by two-way ANOVA (material group, time) with Tukey’s post hoc test. An external SEM-based qualitative laboratory report was used as complementary documentation of *C. albicans* presence after 1 h and 24 h; representative micrographs and quantitative SEM image outputs were unavailable. **Results:** Material group, time, and their interaction significantly affected adhered *C. albicans* counts (*p* < 0.05). At 1 h, the comparator pattern resin showed the highest adhesion, whereas at 24 h, milled CAD/CAM PMMA showed the highest adhered load. For the adhered/inoculum fraction, both material group and time were significant; at 24 h, the heat-polymerized acrylic resin showed the lowest adhered fraction. **Conclusions:** Under the standardized finishing and mechanical polishing conditions of this in vitro model, the tested material groups showed different *C. albicans* adhesion/biofilm patterns over time; clinical extrapolation should be made with caution.

## 1. Introduction

Denture stomatitis is one of the most frequent complications in removable denture wearers, with a global prevalence estimated between 20 and 67%, and it is particularly relevant in aging populations with multiple comorbidities [1]. Its onset is mainly linked to colonization of acrylic denture-base surfaces by yeasts of the genus *Candida*, especially *C. albicans*, and to biofilm maturation on the denture base [2]. In older adults, dentures can harbor substantial loads of opportunistic respiratory pathogens, reinforcing the preventive role of adequate hygiene and biofilm control in the oral cavity [3].

The incorporation of digital workflows has expanded the range of materials and fabrication techniques, including heat-polymerized, autopolymerizing, microwave-polymerized, and milled CAD/CAM resins, as well as photopolymerized 3D-printed resins, each with surface properties that condition *C. albicans* adhesion and biofilm organization [4,5]. Overall, in vitro evidence and recent reviews indicate a general trend toward lower adhesion on milled PMMA and, in many scenarios, on heat-polymerized acrylics compared with certain 3D-printed resins [2,6,7]. However, findings are not uniform: differences have been reported between brands and systems, with inconsistent correlations with surface roughness and contact angle [8,9], and printing parameters such as layer thickness or build angle modify the topography, with effects that may or may not translate into measurable changes in microbial adhesion [10,11].

This heterogeneity is compounded by protocol differences (degree of polishing, post-curing, media and incubation times), which hinder comparisons across studies and limit mechanistic inferences about the role of fabrication technique in fungal colonization [4,5]. In addition, hygiene procedures, both chemical and mechanical, can alter roughness and biofilm load, with particularly marked effects in some printed resins [12,13,14]. Even in the clinical setting, classifying a denture as “clean” or “not clean” is associated with distinct microbiological profiles, highlighting the need for more standardized experimental conditions [3].

In addition to initial adhesion, Candida albicans colonization of denture-base materials is clinically relevant because fungal cells may be retained within surface defects, porosities, and microirregularities, potentially reducing the effectiveness of routine cleansing and facilitating recurrent biofilm formation. In PMMA-based materials, this phenomenon may be influenced by processing-related characteristics, including polymerization method, residual monomer content, internal porosity, and surface homogeneity, all of which can affect microorganism–substrate interactions. Furthermore, persistent microbial colonization and biofilm-associated metabolites have been linked to surface deterioration and may contribute to unfavorable changes in selected physical and mechanical properties over time [15,16]. Therefore, comparing C. albicans adhesion among denture-base-related material groups processed using different techniques is relevant not only for microbiological risk assessment but also for understanding how processing-dependent material characteristics may influence long-term clinical performance.

Although the body of evidence agrees that surface properties and processing influence Candida colonization [2], few studies have compared different fabrication-related material groups within a single temporal and quantitative framework. Therefore, this study aimed to compare early adhesion (1 h) and initial biofilm formation (24 h) of Candida albicans across five material groups, including one comparator pattern resin group and four denture-base resin groups (heat-polymerized acrylic resin, autopolymerizing acrylic resin, microwave-polymerized acrylic resin, and milled CAD/CAM PMMA), using adhered cell counts and adhered/inoculum ratios as outcomes. We hypothesized that material group and incubation time would differentially modulate both processes.

## 2. Materials and Methods

### 2.1. Study Design and Sample

An in vitro experimental study with a 5 × 2 factorial design was conducted, considering material group (five levels) and incubation time (1 h and 24 h) as factors. The primary outcome was the count of Candida albicans cells adhered to the denture base specimens, expressed as colony-forming units per milliliter (CFU/mL). As a secondary outcome, the proportion of adhered cells relative to the initial inoculum (adhered/inoculum) was evaluated.

Acrylic discs measuring 10 mm in diameter and 2 mm in thickness were fabricated from five acrylic resin material groups relevant to denture-base fabrication workflows: M01, an autopolymerizing acrylic pattern resin used as a comparator material (DuraLay Inlay Pattern Resin, Reliance Dental Mfg. Co., Alsip, IL, USA); M02, a heat-polymerized PMMA denture-base resin (SR Triplex Hot, Ivoclar Vivadent AG, Schaan, Liechtenstein); M03, an autopolymerizing PMMA denture-base resin (AESTHETIC BLUE, CANDULOR AG, Zurich, Switzerland); M04, a pre-polymerized CAD/CAM PMMA milling blank (HappyZir Mono-layer PMMA Blank, Hunan Vsmile Biotechnology Co., Ltd., Changsha, China); and M05, a microwave-polymerized PMMA denture-base resin (Acron MC, GC Corporation, Tokyo, Japan). For each material group, 14 discs were prepared for the 1 h time point and 14 for the 24 h time point, yielding a total of 140 specimens (5 material groups × 2 time points × 14 replicates). Additional discs were fabricated for external qualitative SEM-based laboratory documentation. The number of specimens per experimental cell (*n* = 14) was defined to provide a balanced factorial design (5 material groups × 2 time points) with sufficient replication for microbiological quantification and between-group comparisons, following sample sizes commonly used in comparable in vitro adhesion/biofilm studies and considering laboratory feasibility and material availability. No a priori formal power analysis was performed. Lot numbers were requested from laboratory records but were not available at the time of manuscript revision and therefore could not be reported. M01 (pattern resin) was intentionally included as a non-definitive comparator material to provide an internal reference for early adhesion and biofilm behavior under the same experimental workflow, and not as a clinical denture-base material for direct practice recommendations.

### 2.2. Specimen Preparation

To improve reproducibility, the specimen fabrication workflow and surface handling procedures are described in detail below. For the flask-processed groups (M01, M02, M03, and M05), disc-shaped patterns (10 mm in diameter × 2 mm in thickness) were standardized using a master template to ensure uniform dimensions. The patterns were invested in conventional dental flasks using Type III dental stone (Labstone, Garreco™ Dental Lab Products, Heber Springs, AR, USA) to obtain mold cavities of standardized dimensions. After stone setting, the molds were opened and the corresponding acrylic materials were processed according to the protocol established for each group.

For M02 (heat-polymerized PMMA), the material was mixed and packed into the mold cavities and polymerized using a conventional boiling water-bath cycle at 100 °C for 45 min. For M03 (autopolymerizing PMMA), the material was mixed and placed into the mold cavities and polymerized under routine laboratory conditions using pressure-pot polymerization (approximately 2 bar and 40–45 °C for 15–20 min; ambient handling temperature approximately 23–25 °C). For M05 (microwave-polymerized PMMA), the material was processed in a special non-metallic microwave flask (fiber-reinforced epoxy resin) using a microwave cycle of 500 W for 3 min. M01 corresponded to an autopolymerizing acrylic pattern resin comparator and was prepared using the same disc dimensions and subsequent surface standardization procedures as the other non-milled groups.

For M04, specimens were obtained by milling disc-shaped samples (10 mm × 2 mm) from pre-polymerized CAD/CAM PMMA blanks using a Roland DWX-52D milling unit (Roland DG Corporation, Hamamatsu, Japan) and the corresponding laboratory CAM workflow. No gypsum mold was used for the CAD/CAM group.

After deflasking (where applicable) and specimen retrieval, all discs underwent standardized finishing and surface handling. Excess material/flash was removed using a tungsten carbide bur, and the surfaces were finished/polished using the same sequence for all groups by mechanical polishing: silicon carbide abrasive papers (320, 600, and 1200 grit) under water irrigation, followed by pumice and polishing paste. Polishing time was approximately 1–2 min per abrasive paper grit, 2–3 min with pumice, and 1–2 min with polishing paste. The surface selected for microbiological testing (test surface) was standardized across specimens by applying the same finishing/polishing protocol and orientation criteria. Specimens were then rinsed with distilled water, ultrasonically cleaned for 5 min, air-dried, and stored in sterile containers until microbiological testing. This standardized post-processing protocol was used to minimize variability in surface condition attributable to mold preparation and specimen handling. Surface roughness and surface free energy were not instrumentally measured in this study. All specimens were evaluated after standardized finishing and mechanical polishing; unpolished surfaces were not tested in this study.

### 2.3. Microbial Strain and Culture Conditions

The reference strain *Candida albicans* ATCC 10231 was used, obtained from an accredited supplier and characterized by MALDI-TOF mass spectrometry and by tests confirming chlamydospore and germ tube formation. The yeast was reactivated on Potato Dextrose Agar and maintained on Sabouraud dextrose agar and broth (SDB) supplemented with 5% sucrose, incubated aerobically at 37 °C for 24 h prior to testing.

### 2.4. Adhesion and Biofilm Formation Model

Before inoculation, discs were sterilized by immersion in 70% ethanol, followed by exposure to ultraviolet light for 30 min, and then placed at the bottom of 20-well microtiter plates, one disc per well.

The microbial suspension was adjusted to 0.5 McFarland units (≈10^6^ cells/mL) using spectrophotometry. Each well received 1.5 mL of this suspension in SDB with sucrose, and plates were incubated at 37 °C for 1 h to assess early adhesion or for 24 h with orbital shaking at 150 rpm to promote initial biofilm formation. Wells containing only sterile SDB supplemented with sucrose were used as negative controls.

At the end of each incubation period (1 h and 24 h), discs were carefully removed and rinsed with 0.1 M phosphate-buffered saline (PBS) to eliminate non-adherent planktonic cells.

### 2.5. Quantification of Adhesion and Calculation of Ratios

Quantification of *C. albicans* was performed by plate counting. For each replicate, the laboratory recorded two values: (i) the concentration of the initial culture immediately before the adhesion assay (inoculum CFU/mL) and (ii) the count of adhered cells (CFU/mL) after incubation.

To obtain the adhered cell count, each rinsed disc was transferred to a sterile tube containing buffer solution, and the cells were detached by vortex agitation followed by sonication in an ultrasonic bath. The resulting suspension was serially diluted in physiological saline and plated on Sabouraud dextrose agar, which was incubated at 37 °C for 24–48 h under aerobic conditions. The number of colonies was recorded, and the adhered cell count (CFU/mL) was calculated for each specimen (primary outcome).

The proportion of adhered cells relative to the inoculum (secondary outcome) was calculated by pairing, for each replicate, the adhered count with its corresponding initial culture according to the following ratio:

adhered/inoculum ratio = adhered CFU/mL/initial CFU/mL

Each ratio was treated as an independent experimental unit for statistical analysis.

### 2.6. Scanning Electron Microscopy (SEM)-Based External Qualitative Documentation

As complementary qualitative documentation, a subset of specimens was submitted to an accredited external laboratory for detection of Candida albicans by scanning electron microscopy (SEM), according to the laboratory report issued by Scientific Quality S.A.C. (Informe de Ensayo Nº SQ251001.02). The report identified the study groups (M01–M05) and documented the qualitative presence of *C. albicans* after 1 h and 24 h fungal exposure. According to the report, two units were processed per sample group. However, the external laboratory report did not include representative micrographs, SEM acquisition parameters, or quantitative image analysis outputs. Therefore, in the present study, the SEM component was limited to external qualitative confirmation of microorganism presence and was not used for comparative morphologic interpretation. The laboratory report is provided as Supplementary File S1.

### 2.7. Statistical Analysis

Statistical analysis was performed using R software, version 4.4.1 (R Foundation for Statistical Computing). For the primary outcome (adhered cell count, CFU/mL), data were log_10_-transformed and analyzed using a two-way ANOVA with material (five levels) and incubation time (two levels) as factors, including the Material Group × Time interaction. When significant effects were detected, post hoc pairwise comparisons were conducted using Tukey’s honestly significant difference (HSD) test.

For the secondary outcome (adhered/inoculum ratio), the ratio was calculated for each replicate and its log_10_ transformation was analyzed with an analogous two-way ANOVA (Material Group × Time), followed by Tukey HSD where appropriate. All analyses were two-sided; 95% confidence intervals and partial eta-squared (η^2^_p_) effect sizes were reported, and statistical significance was set at *p* < 0.05.

## 3. Results

Descriptive statistics for absolute *Candida albicans* counts are presented in Table 1. At 1 h, the comparator pattern resin showed the highest fungal load (median ≈ 190,000 CFU/mL; IQR 150,000–287,500), clearly above the other tested groups, whose counts remained below 60,000 CFU/mL (e.g., heat-polymerized acrylic resin group with a median ≈ 27,000 CFU/mL). At 24 h, counts increased for most materials, with milled CAD/CAM PMMA (median ≈ 115,000 CFU/mL) and the comparator pattern resin (≈99,500 CFU/mL) showing the highest values, while the heat-polymerized acrylic resin group maintained the lowest counts (≈39,500 CFU/mL). The two-way ANOVA on log_10_(CFU/mL) (Table 2) showed significant effects of material group (*p* < 0.001; partial η^2^ = 0.357) and incubation time (*p* = 0.003; partial η^2^ = 0.065), as well as a significant Material group × Time interaction (*p* < 0.001; partial η^2^ = 0.268), indicating that the increase in fungal load from 1 h to 24 h depended on the tested group. Tukey HSD comparisons (Table 3) revealed that, at 1 h, the comparator pattern resin had significantly higher counts than all other tested groups (adjusted *p* < 0.001 in most pairwise contrasts), and that at 24 h milled CAD/CAM PMMA showed higher counts than the heat-polymerized, autopolymerizing, and microwave-polymerized acrylic resins groups (adjusted *p* ≤ 0.032), while the comparator pattern resin maintained higher values than the heat-polymerized acrylic resin group (*p* = 0.001).

The proportions of the inoculum that remained adhered are shown in Table 4. The comparator pattern resin exhibited the highest fractions at both 1 h (median ≈ 0.48) and 24 h (≈0.43), followed by milled CAD/CAM PMMA (≈0.35 and 0.40, respectively). In contrast, the heat-polymerized acrylic resin group showed the lowest adhered proportions, particularly at 24 h (median ≈ 0.15), reflecting a lower relative capacity to retain fungal cells under the conditions of this model. The two-way ANOVA for log_10_(adhered/inoculum) (Table 5) confirmed significant effects of material group (*p* < 0.001; partial η^2^ = 0.254) and time (*p* = 0.032; partial η^2^ = 0.035), as well as a Material Group × Time interaction (*p* = 0.039; partial η^2^ = 0.074). According to Tukey comparisons (Table 6), at 1 h the comparator pattern resin and milled CAD/CAM PMMA showed significantly higher adhered proportions than the microwave-polymerized acrylic resin group, whereas at 24 h the comparator pattern resin exceeded both the heat-polymerized and microwave-polymerized acrylic resin groups (adjusted *p* ≤ 0.01). Conversely, at 24 h, the heat-polymerized acrylic resin exhibited significantly lower proportions than the autopolymerizing acrylic resin, milled CAD/CAM PMMA, and microwave-polymerized acrylic resin groups (adjusted *p* ≤ 0.022), supporting its characterization as the least favorable group for *C. albicans* adhesion among the denture-base resin groups in this model.

### External Qualitative SEM-Based Documentation

According to the external laboratory SEM-based report, Candida albicans was qualitatively reported as present in all tested material groups (M01–M05) after both 1 h and 24 h fungal exposure. Because the report did not provide representative micrographs or quantitative SEM image outputs, no intergroup comparison of surface morphology, microorganism distribution, or structural forms was performed from the SEM component.

## 4. Discussion

In this in vitro model, the pattern resin (M01) showed the highest early adhesion at 1 h, whereas at 24 h, the greatest fungal load was observed on milled CAD/CAM PMMA (M04), and the heat-polymerized acrylic resin (M02) acted as the material with the lowest adhered fraction of the inoculum. This pattern suggests that the surface characteristics of M01 may favor initial colonization, whereas the higher fungal load observed at 24 h on M04 may be related to physicochemical properties of pre-polymerized PMMA and/or to processing- and finishing-related variables not directly measured in this study [17,18]. The observed Material × Time interaction is consistent with recent studies indicating that the transition from initial adhesion to a more established biofilm depends on surface characteristics, material aging/use, and the post-processing applied [19,20,21].

Because M01 was included as a comparator material rather than a definitive denture-base material, its performance should be interpreted primarily as an internal reference within the experimental model rather than as a basis for direct clinical material selection.

The literature indicates that, on average, CAD/CAM-milled denture bases exhibit lower roughness than 3D-printed and conventionally processed bases, which has been interpreted as a potential advantage against microbial colonization [17]. However, studies specifically focused on *C. albicans* report heterogeneous findings: some describe greater colonization on printed surfaces than on milled ones [19], others report similar susceptibility between printed resins and heat-polymerized PMMA [22], and in clinical or pilot settings, clear differences between fabrication techniques are not always detected [23,24]. This variability has been linked to processing parameters such as print orientation, post-curing, and toothbrushing-induced wear, which modify topography and may increase biofilm formation even on initially smooth resins [21]. Likewise, chemical polishing has been shown to reduce sorption, surface free energy, and *C. albicans* adhesion compared with mechanical polishing, even without appreciable changes in mean roughness, underscoring the role of additional surface variables beyond roughness alone [25]. In parallel, several antimicrobial modification strategies, such as incorporation of TiF_4_ [26], zinc-modified phosphate glass microfiller in autopolymerizing resin [27], embedded micro/nanoparticles [28], or AgVO_3_ in PMMA [29], have demonstrated reductions in CFU counts or biofilm activity, although in some cases at the expense of mechanical or structural properties [28,30].

The present study contributes a factorial design that distinguishes between early adhesion (1 h) and initial biofilm formation (24 h), with an adequate number of replicates and complementary external qualitative SEM-based documentation of microorganism presence. A limitation of the SEM component is that the external laboratory report documented only qualitative presence of C. albicans and did not include representative micrographs, SEM acquisition parameters, or quantitative image outputs; therefore, no image-based morphologic comparison among materials could be performed. However, the study is also limited to two time points, a single fungal species (*C. albicans*), and the absence of direct measurements of roughness, contact angle, or surface free energy—key parameters for interpreting differences among materials [17,18]. Therefore, any interpretation relating intergroup differences to surface topography or physicochemical behavior should be considered hypothesis-generating and not a direct measurement-based conclusion. In addition, finishing/polishing and post-processing conditions (layering, build orientation, post-curing) could not be fully standardized in the external laboratory, which may have contributed to the greater biofilm observed at 24 h on M04 despite the body of evidence that, in general, favors milled surfaces over other technologies [19,21,22].

These findings should be interpreted within the context of a standardized in vitro finishing and mechanical polishing protocol and should not be extrapolated as evidence of absolute clinical superiority of one material over another. From a clinical perspective, these findings reinforce that the quality of finishing and maintenance may be as decisive as the fabrication technique itself, since appropriate polishing and hygiene protocols reduce adhesion and sorption [21,25]; that milled denture bases, although typically exhibiting more favorable surface characteristics, may still support mature biofilms if altered by use, wear, and other surface-related factors, making both clinical and home care indispensable [17,19]; and that incorporating antimicrobial additives such as TiF_4_, bioactive glasses, or microparticles represents a promising option for high-risk patients, but requires clinical validation and a detailed assessment of their mechanical impact and implications for the fabrication process [24,25,26,27,28].

## 5. Conclusions

Within the limitations of this in vitro study, the following conclusions can be drawn:

• The denture base fabrication technique significantly modulates early adhesion and initial biofilm formation of *Candida albicans*, with a clear Material × Time interaction.
• In this model, under standardized finishing and mechanical polishing conditions, the comparator pattern resin (M01) exhibited the highest adhesion at 1 h, whereas milled CAD/CAM PMMA (M04) showed the highest adhered fungal load at 24 h, and the heat-polymerized acrylic resin (M02) showed the lowest adhered fraction of the inoculum, based on the quantitative microbiological outcomes of this model.
• Both material selection and surface management appear to be relevant determinants of biofilm-related risk on denture bases.
• These results provide a useful comparative in vitro framework to inform material selection and to prioritize finishing and maintenance strategies in patients at risk of denture stomatitis, although clinical extrapolation should be made with caution.

## Figures and Tables

**Table 1:** Adhesion of *Candida albicans* by material and incubation time (CFU/mL).

Material	Time	n	Median [IQR] (CFU/mL)	Geometric Mean (CFU/mL)	log_10_ (Geomean)
Auto-polymerized acrylic resin	1 h	14	55,000 [43,500, 66,500]	53,767	4.731
Microwave-polymerized acrylic resin	1 h	14	41,500 [20,250, 56,000]	25,096	4.4
Heat-polymerized acrylic resin	1 h	14	27,000 [22,250, 33,000]	27,345	4.437
PMMA (CAD/CAM milled)	1 h	14	34,000 [23,250, 43,750]	31,943	4.504
Pattern resin	1 h	14	190,000 [150,000, 287,500]	182,907	5.262
Auto-polymerized acrylic resin	24 h	14	55,000 [47,750, 70,250]	57,975	4.763
Microwave-polymerized acrylic resin	24 h	14	50,500 [40,250, 69,250]	53,196	4.726
Heat-polymerized acrylic resin	24 h	14	39,500 [30,250, 43,750]	37,017	4.568
PMMA (CAD/CAM milled)	24 h	14	115,000 [100,000, 147,500]	128,688	5.11
Pattern resin	24 h	14	99,500 [42,500, 147,500]	75,955	4.881

IQR, interquartile range (p25–p75); CFU, colony-forming units; the geometric mean corresponds to the antilogarithm of the mean of log_10_-transformed values; n, number of replicates per material group and time point.

**Table 2:** Two-way ANOVA of *Candida albicans* load (log_10_[CFU/mL]) according to material group and incubation time.

Effect	df	F	*p*	Partial η^2^
Material	4	18.028	<0.001	0.357
Time	1	9.096	0.003	0.065
Material × Time	4	11.926	<0.001	0.268

Dependent variable: log_10_(CFU/mL) of *Candida albicans*. Model: two-way ANOVA with material group and incubation time, including the Material Group × Time interaction df: degrees of freedom; partial η^2^: partial effect size.

**Table 3:** Multiple pairwise comparisons (Tukey HSD) of *Candida albicans* load (log_10_[CFU/mL]) according to material group and incubation time—pairs with significant differences.

Time	Group 1	Group 2	Mean Difference (log_10_)	95% CI of the Difference	Adjusted *p*
1 h	Pattern resin	Heat-polymerized acrylic resin	−0.825	[−1.186, −0.465]	<0.001
1 h	Pattern resin	Auto-polymerized acrylic resin	−0.532	[−0.893, −0.171]	0.001
1 h	Pattern resin	PMMA (CAD/CAM)	−0.758	[−1.119, −0.397]	<0.001
1 h	Pattern resin	Microwave-polymerized acrylic resin	−0.863	[−1.224, −0.502]	<0.001
24 h	Pattern resin	Heat-polymerized acrylic resin	−0.312	[−0.527, −0.097]	0.001
24 h	Pattern resin	PMMA (CAD/CAM)	0.229	[0.014, 0.444]	0.032
24 h	Heat-polymerized acrylic resin	PMMA (CAD/CAM)	0.541	[0.326, 0.756]	<0.001
24 h	Auto-polymerized acrylic resin	PMMA (CAD/CAM)	0.346	[0.131, 0.562]	<0.001
24 h	PMMA (CAD/CAM)	Microwave-polymerized acrylic resin	−0.384	[−0.599, −0.169]	<0.001

Post hoc Tukey HSD test based on the two-way ANOVA model. Dependent variable: log_10_(CFU/mL). Mean difference is calculated as Group 1 − Group 2. 95% CI: 95% confidence interval of the difference. Adjusted *p*: *p*-value adjusted for multiple comparisons. Only comparisons with adjusted *p* < 0.05 are shown.

**Table 4:** Adhered/inoculum ratio of Candida albicans by material group and incubation time.

Material	Time	n	Median [IQR]	Geometric Mean
Auto-polymerized acrylic resin	1 h	14	0.20 [0.17, 0.32]	0.227
Microwave-polymerized acrylic resin	1 h	14	0.19 [0.10, 0.35]	0.143
Heat-polymerized acrylic resin	1 h	14	0.21 [0.16, 0.28]	0.215
PMMA (CAD/CAM)	1 h	14	0.35 [0.26, 0.41]	0.314
Pattern resin	1 h	14	0.48 [0.31, 0.67]	0.453
Auto-polymerized acrylic resin	24 h	14	0.39 [0.28, 0.53]	0.382
Microwave-polymerized acrylic resin	24 h	14	0.26 [0.19, 0.37]	0.267
Heat-polymerized acrylic resin	24 h	14	0.15 [0.12, 0.20]	0.166
PMMA (CAD/CAM)	24 h	14	0.40 [0.32, 0.45]	0.389
Pattern resin	24 h	14	0.43 [0.33, 0.73]	0.451

Adhered/inoculum ratio = adhered CFU/mL/initial inoculum CFU/mL. IQR: interquartile range (p25–p75); the geometric mean corresponds to the antilogarithm of the mean of log_10_-transformed values; n: number of replicates per material group and time point.

**Table 5:** Two-way ANOVA of the adhered/inoculum (log10[adhered//inoculum]) according to material and incubation time.

Effect	df	F	*p*	Partial η^2^
Material	4	11.091	<0.001	0.254
Time	1	4.695	0.032	0.035
Material × Time	4	2.605	0.039	0.074

Dependent variable: log10(adhered//inoculum) ratio of *Candida albicans*. Model: two-way ANOVA with factors material group, incubationtime, including the Material × Time interaction. df: degrees of freedom; partial η^2^: partial effect size.

**Table 6:** Multiple comparisons (Tukey HSD) of the adhered/inoculum ratio (log10[adhered/inoculum]) according to material group and incubation time (significant pairs only).

Time	Group 1	Group 2	Mean Difference (log10)	95% CI of the Difference	Adjusted *p*
1 h	Pattern resin	Microwave-polymerized acrylic resin	−0.499	[−0.840, −0.158]	0.001
1 h	PMMA (CAD/CAM)	Microwave-polymerized acrylic resin	−0.341	[−0.681, −0.000]	0.05
24 h	Pattern resin	Heat-polymerized acrylic resin	−0.434	[−0.621, −0.247]	<0.001
24 h	Pattern resin	Microwave-polymerized acrylic resin	−0.227	[−0.414, −0.039]	0.01
24 h	Heat-polymerized acrylic resin	Auto-polymerized acrylic resin	0.362	[0.175, 0.55]	<0.001
24 h	Heat-polymerized acrylic resin	PMMA (CAD/CAM)	0.37	[0.183, 0.558]	<0.001
24 h	Heat-polymerized acrylic resin	Microwave-polymerized acrylic resin	0.208	[0.02, 0.395]	0.022

Post hoc Tukey HSD post hoc test based on the two-way ANOVA model. Dependent variable: log10(adhered/inoculum) ratio. The mean difference is calculated as Group 1 − Group 2. 95% CI: 95% confidence interval of the difference; adjusted *p*: *p* value adjusted for multiple comparisons. Only comparisons with adjusted *p* < 0.05 are shown.

## Data Availability

The data are not publicly available due to institutional policy regarding laboratory datasets. The data presented in this study are available on reasonable request from the corresponding author.

## Supplementary Materials

The following supporting information can be downloaded at: https://www.mdpi.com/article/10.3390/dj14050262/s1, File S1: laboratory report.
