# Supplementary material for: Influence of Denture Base Fabrication on Candida albicans Adhesion and Early Biofilm: An In Vitro Comparison of Five Techniques
Source: Dent J (Basel). 2026 May 1;14(5):262. doi: 10.3390/dj14050262 (PMC13205908; doi:10.3390/dj14050262)
Supplement: Supplementary file 1 [file dentistry-14-00262-s001.zip › dentistry-4154107-supplementary.pdf]

INFORME DE ENSAYO Nº SQ251001.02

SOLICITUD DE ENSAYO	:	SQE 250915.01
SOLICITANTE	:	Universidad Privada Norbert Wiener
DIRECCIÓN DEL SOLICITANTE	:	No indica
PROCEDENCIA DE LA MUESTRA	:	Proporcionado por el solicitante.
IDENTIFICACIÓN DE LA MUESTRA	:	M01: Discos de 2mm por 10mm Maqueta Resina<br>M02: Discos de 2mm por 10mm Maqueta AC Termo<br>M03: Discos de 2mm por 10mm Maqueta AC Autocurado<br>M04: Discos de 2mm por 10mm Maqueta PMMA<br>M05: Discos de 2mm por 10mm Maqueta AC Microondas
CANTIDAD Y DESCRIPCIÓN DE LA MUESTRA	:	M01, M02, M03, M04, M05: Dos (02) unidades por muestra.
FECHA Y HORA DE RECEPCIÓN	:	16 de septiembre del 2025/ 10:30h
CONDICIONES A LA RECEPCIÓN	:	Temperatura ambiente
FECHAS DE INICIO DEL ANÁLISIS	:	16 de septiembre del 2025
FECHAS DE TÉRMINO DEL ANÁLISIS	:	01 de octubre del 2025
FECHAS DE EMISIÓN	:	01 de octubre del 2025

RESULTADOS DE ENSAYO MICROBIOLÓGICO

Exposición fúngica de 1 hora de los discos frente a Candida albicans ATCC 10231				
**M01**	**M02**	**M03**	**M04**	**M05**
Presencia de *Candida albicans*	Presencia de *Candida albicans*	Presencia de *Candida albicans*	Presencia de *Candida albicans*	Presencia de *Candida albicans*

V°B° ÁREA DE LABORATORIO — SCIENTIFIC QUALITY S.A.C.

Exposición fúngica de 24 horas de los discos frente a Candida albicans ATCC 10231.				
**M01**	**M02**	**M03**	**M04**	**M05**
Presencia de *Candida albicans*	Presencia de *Candida albicans*	Presencia de *Candida albicans*	Presencia de *Candida albicans*	Presencia de *Candida albicans*

MÉTODOS DE ENSAYO	
ENSAYOS	REFERENCIA
Detección de *Candida albicans*	Microscopia electronica de barrido (SEM)

SCIENTIFIC QUALITY S.A.C. — JEFATURA DE LABORATORIO

Mblgo. Oniel Elías Juarez Vilcapuma
Jefe de Laboratorio
C.B.P.14090

Los resultados de los ensayos corresponden solo a la(s) muestra(s) ensayada(s). Los resultados no deben ser utilizados como una certificación de conformidad con normas de producto o como certificado del sistema de calidad de la entidad que lo produce. Queda prohibida la reproducción parcial o total del presente informe, sin la autorización escrita por SCIENTIFIC QUALITY S.A.C, la adulteración o uso indebido del presente informe constituye un delito contra la fe pública y se regula por las disposiciones penales y civiles en la materia.

Dirección: Mz. N Lote 1. Barrio 4. Sector 2. Parcela-Subparcela B. Agrup. Pachacamac. Villa El Salvador. Lima.
Celular: 999 365 647. Correo electrónico: laboratorio@scientificquality.org. Web: www.scientificquality.org
